# Evaluation of safety and efficacy of inhaled ambroxol in hospitalized adult patients with mucopurulent sputum and expectoration difficulty

**DOI:** 10.3389/fmed.2023.1182602

**Published:** 2023-05-25

**Authors:** Zeguang Zheng, Kai Yang, Ni Liu, Xiuhua Fu, Huijie He, Hong Chen, Peijun Xu, Jing Wang, Maofeng Liu, Yuling Tang, Fengzi Zhao, Shufeng Xu, Xiaowei Yu, Jichang Han, Bo Yuan, Bin Jia, Guifen Pang, Yantong Shi, Min Kuang, Haiyan Shao, Hao Xiong, Jia He, Yuanyuan Pan, Rongchang Chen

**Affiliations:** ^1^State Key Laboratory of Respiratory Disease, National Clinical Research Center for Respiratory Disease, Guangzhou Institute of Respiratory Health, The First Affiliated Hospital of Guangzhou Medical University, Guangzhou, China; ^2^Shenzhen Institute of Respiratory Disease, Shenzhen People's Hospital (First Affiliated Hospital of South University of Science and Technology and Second Affiliated Hospital of Jinan University), Shenzhen, China; ^3^The Affiliated Hospital of Inner Mongolia Medical University, Hohhot, China; ^4^The First Affiliated Hospital of Baotou Medical College Inner Mongolia University of Science and Technology, Baotou, China; ^5^The Second Affiliated Hospital of Harbin Medical University, Harbin, China; ^6^Zibo Central Hospital, Zibo, China; ^7^The First Affiliated Hospital of Zhengzhou University, Zhengzhou, China; ^8^PKU Care Luzhong Hospital, Zibo, China; ^9^The First Hospital of Changsha, Changsha, China; ^10^The First Hospital of Qiqihar, Qiqihar, China; ^11^First Hospital of Qinhuangdao, Qinhuangdao, China; ^12^Changzhou No.2 People's Hospital, Changzhou, China; ^13^Huaihe Hospital of Henan University, Kaifeng, China; ^14^Siping Central People's Hospital, Siping, China; ^15^The First Affiliated Hospital of Xinjiang Medical University, Urumqi, China; ^16^Affiliated Hospital of Chengde Medical University, Chengde, China; ^17^People's Hospital of Rizhao, Rizhao, China; ^18^The Second Nanning People's Hospital, Nanning, China; ^19^The First People's Hospital of Wenling, Wenling, China; ^20^The Second People's Hospital of Yibin, Yibin, China; ^21^Department of Health Statistics, Faculty of Medical Service, Naval Medical University, Shanghai, China; ^22^Yingu Pharmaceutical Co Ltd., Beijing, China

**Keywords:** inhaled ambroxol, lower respiratory tract infection, mucopurulent sputum, expectoration difficulty, safety, efficacy

## Abstract

**Background:**

Ambroxol is a widely used mucoactive drug in sputum clearance of respiratory diseases taken orally and by injection. However, there is a paucity of evidence for inhaled ambroxol in sputum clearance.

**Methods:**

This study performed a multicenter, randomized, double-blind, placebo-controlled, phase 3 trial at 19 centers in China. Hospitalized adult patients with mucopurulent sputum and expectoration difficulty were recruited. Patients were randomized by 1:1 to receive inhalation of either ambroxol hydrochloride solution 3 mL (22.5 mg) + 0.9% sodium chloride 3 mL or 0.9% sodium chloride 6 mL twice daily for 5 days, with an interval of more than 6 h. The primary efficacy endpoint was the absolute change in the sputum property score after treatment compared to the baseline in the intention-to-treat population.

**Results:**

Between 10 April 2018 and 23 November 2020, 316 patients were recruited and assessed for eligibility, of whom 138 who received inhaled ambroxol and 134 who received a placebo were included. Patients who received inhaled ambroxol had a significantly greater decrease in the sputum property score compared with patients who received inhalation of placebo (difference: −0.29; 95% CI: −0.53 to −0.05; *p* = 0.0215). Compared with the placebo, inhaled ambroxol also significantly reduced more expectoration volume in 24 h (difference: −0.18; 95% CI: −0.34 to −0.03; *p*  = 0.0166). There was no significant difference in the proportion of adverse events between the two groups, and no deaths were reported.

**Discussion:**

In hospitalized adult patients with mucopurulent sputum and expectoration difficulty, inhaled ambroxol was safe and effective for sputum clearance compared with a placebo.

**Clinical trial registration:**

[https://www.chictr.org.cn/showproj.html?proj=184677], Chinese Clinical Trial Registry [ChiCTR2200066348].

## 1. Introduction

Mucopurulent sputum and expectoration difficulty are common symptoms of respiratory diseases, which are likely associated with lower respiratory tract infections (LRTIs, including pneumonia and acute bronchitis) and acute exacerbation of chronic obstructive pulmonary disease (COPD) or bronchiectasis (1, 2). Increased sputum and expectoration difficulty exacerbate cough, expectoration, chest tightness, dyspnea, and other symptoms, which may lead to treatment failure (3, 4). In serious cases, excessive high-viscosity sputum is an important cause of unexpected death due to suffocation (5, 6). Therefore, in addition to the etiological treatment, sputum clearance by physiotherapies or mucoactive drugs can alleviate symptoms, shorten hospital stays, and improve prognosis (7–9).

Mucoactive drugs are commonly used to clear the airway in mucus hypersecretory diseases, which can alleviate mucus hypersecretion and increase the efficiency of expectoration (10). Theoretically, inhaled medications can be directly and rapidly delivered to the lower respiratory tract and exert therapeutic effects, with fewer systemic adverse effects (11, 12). Meanwhile, nebulization can also dilute sputum and lubricate the respiratory tract to reduce respiratory irritation. N-acetylcysteine inhalation is the most widely used inhaled mucoactive drug (13–16). However, high-quality evidence for the inhalation of other mucoactive drugs is still limited.

Ambroxol is a widely used mucoactive drug to treat respiratory diseases associated with abnormal mucus secretion and impaired mucus transport (17). Ambroxol is available in various pharmaceutical forms, including intramuscular solution, intravenous solution, suppository, syrup, granule, tablet, capsule, slow release oral formulation, and nebulized solution (18). The safety and efficacy of some forms have been confirmed in numerous studies (19–22). A previous trial showed that optimized perioperative airway management by inhaled ambroxol could reduce postoperative complications and shorten hospital stays in lung cancer patients (23). Although used clinically, to the best of our knowledge, no randomized double-blind controlled trial has been published to evaluate the safety and efficacy of inhaled ambroxol in adult patients with mucopurulent sputum. In this study, we aimed to investigate the safety and efficacy of inhaled ambroxol in hospitalized adult patients with mucopurulent sputum compared with a placebo.

## 2. Materials and methods

### 2.1. Study design and participants

We performed a multicenter, randomized, double-blind, placebo-controlled trial at 19 study centers in China to assess the safety and efficacy of inhaled ambroxol hydrochloride solution to improve sputum clearance in hospitalized adult patients with mucopurulent sputum and expectoration difficulty. The trial complied with the International Conference on Harmonization Good Clinical Practice guidelines, the Declaration of Helsinki, and all relevant local regulations. The protocol was reviewed and approved by the Ethics Committee of First Affiliated Hospital of Guangzhou Medical University ([2017]-medicine-028-02), and copy of the ethics committee approval was also approved by independent ethics committees of all study sites. Written informed consent was obtained from all study participants before the screening.

The inclusion criteria for participants were as follows: (1) patients aged 18–80 years old without limitation on sex; (2) patients hospitalized due to lower respiratory tract infections (e.g., pneumonia, and acute exacerbation of COPD or bronchiectasis); and (3) patients with mucopurulent sputum (sputum property score ≥ 2 points) and expectoration difficulty (expectoration difficulty ≥2 points; Table 1).

**Table 1:** Scoring criteria for the primary and secondary efficacy endpoints in this study.

Efficacy endpoint	0	1	2	3
Sputum property score	No mucoid sputum	Mucoid sputum	Mucopurulent sputum (purulent <2/3)	Mucopurulent sputum (purulent ≥2/3)
Cough intensity	No cough	Mild cough, without effects on quality of life	Moderate cough, with some effects on work and sleep	Severe cough, seriously affecting work and sleep
Expectoration difficulty	No sputum	Easy	Moderate	Difficult
Expectoration volume in 24 h	<10 mL	10–50 mL	51–100 mL	> 100 mL

The exclusion criteria were as follows: (1) patients who were known to be allergic to the ingredients contained in the study drug; (2) patients with severe respiratory diseases and other severe primary diseases of cardiovascular, cerebrovascular, liver, kidney, and hematopoietic system; (3) patients with alcoholism, drug dependence, and history of epilepsy or mental disorder; (4) pregnant and lactating women; (5) patients with difficulty in coughing up sputum due to tracheal stenosis (e.g., the history of respiratory tract tumor and foreign body airway obstruction); (6) patients who cannot cooperate with inhalation treatment; and (7) patients complicated with hemoptysis. Detailed exclusion criteria were provided in the Supplementary material.

### 2.2. Randomization and masking

Eligible participants were randomized by a ratio of 1:1 to receive inhalation of ambroxol hydrochloride solution or placebo (0.9% sodium chloride). Randomization was done with variable block sizes, and patients were stratified when entering the study by centers. Allocation was concealed with a prespecified computer-generated sequence, which was kept by the main study center. The drugs were prepared before the initiation of the study and packed into identical containers according to the randomization list. All patients used the same inhalation devices [PARI Boy® Sx (085G3005) compressor and PARI LC SPRINT; PARI GmbH, Starnberg, Germany]. Patients, investigators, and all personnel participating in the treatment or clinical evaluation were blinded to treatment assignment.

### 2.3. Procedures

Patients received inhalation of either ambroxol hydrochloride solution 3 mL (22.5 mg) + 0.9% sodium chloride 3 mL or 0.9% sodium chloride 6 mL twice daily for 5 days, with an interval of more than 6 h (Supplementary Table 1). At the baseline visit, eligibility for enrollment, demographic characteristics, and disease and treatment history were assessed. During the treatment period and 24 h after treatment, efficacy and safety evaluation were conducted.

Antibiotics, antipyretics, and drugs for underlying diseases were allowed according to the symptoms and examinations of patients. Short-acting β-2-agonists in the form of an aerosol or dry powder can also be used as needed, with an interval of ≥2 h with a study drug or placebo. However, drugs that can relieve mucopurulent sputum (e.g., antitussives and expectorants), long-acting β-2-agonists, leukotriene receptor antagonists, anticholinergics, and other inhalation drugs that may affect the efficacy evaluation were prohibited 24 h before and throughout the trial period.

### 2.4. Outcomes

The primary efficacy endpoint was the absolute change in the sputum property score from the baseline visit to 24 h after treatment (24). Secondary efficacy endpoints were the absolute change in cough intensity, expectoration difficulty, and expectoration volume in 24 h from the baseline visit to 24 h after treatment. These scores ranged from 0 to 3, in which higher scores indicated more severe symptoms (Table 1). Safety assessments included the number of adverse events and the frequency of clinically significant abnormal changes in vital signs, physical examination, laboratory evaluation (routine blood test, routine urine test, hepatic function, and renal function), and electrocardiogram.

### 2.5. Statistical analysis

Sample size calculation was based on the primary endpoint, considering a difference between the two groups of 0.5 points, a standard deviation of one point, a two-sided α of 5%, and a power of 90%. We estimated that 86 patients in each group were required to demonstrate statistical significance between the two groups. Considering the requirement of regulatory authority for drug approval (at least 100 in each group), the final sample size was 125 patients in each group with a 20% drop-out rate.

Efficacy outcomes were assessed in the intentiontotreat population, which included all randomized patients who received at least one dose of their assigned study medication. Missing data for the efficacy endpoints were imputed with the last observation carried forward. Safety outcomes were assessed using the safety population of all randomly allocated patients, who were exposed to at least one dose of study medication and had at least one post-dose safety assessment.

Descriptive statistics were presented with mean ± SD, median (inter-quartile range), or frequencies (percentages), as appropriate. Categorical variables were compared using the chi-square test or Fisher exact test, and continuous variables were compared using the *t*-test or Wilcoxon test. Efficacy outcomes were mainly compared using the Wilcoxon test. The primary efficacy outcome was also compared using logistic regression, after adjusting for baseline score, center, and interaction between center and treatment. All reported values of *p* were two-sided and considered statistically significant when value of *p* < 0.05. All analyses were performed using SAS software (version 9.4; SAS Institute, Cary, NC, United States).

## 3. Results

### 3.1. Patient characteristics

**Figure 1:**
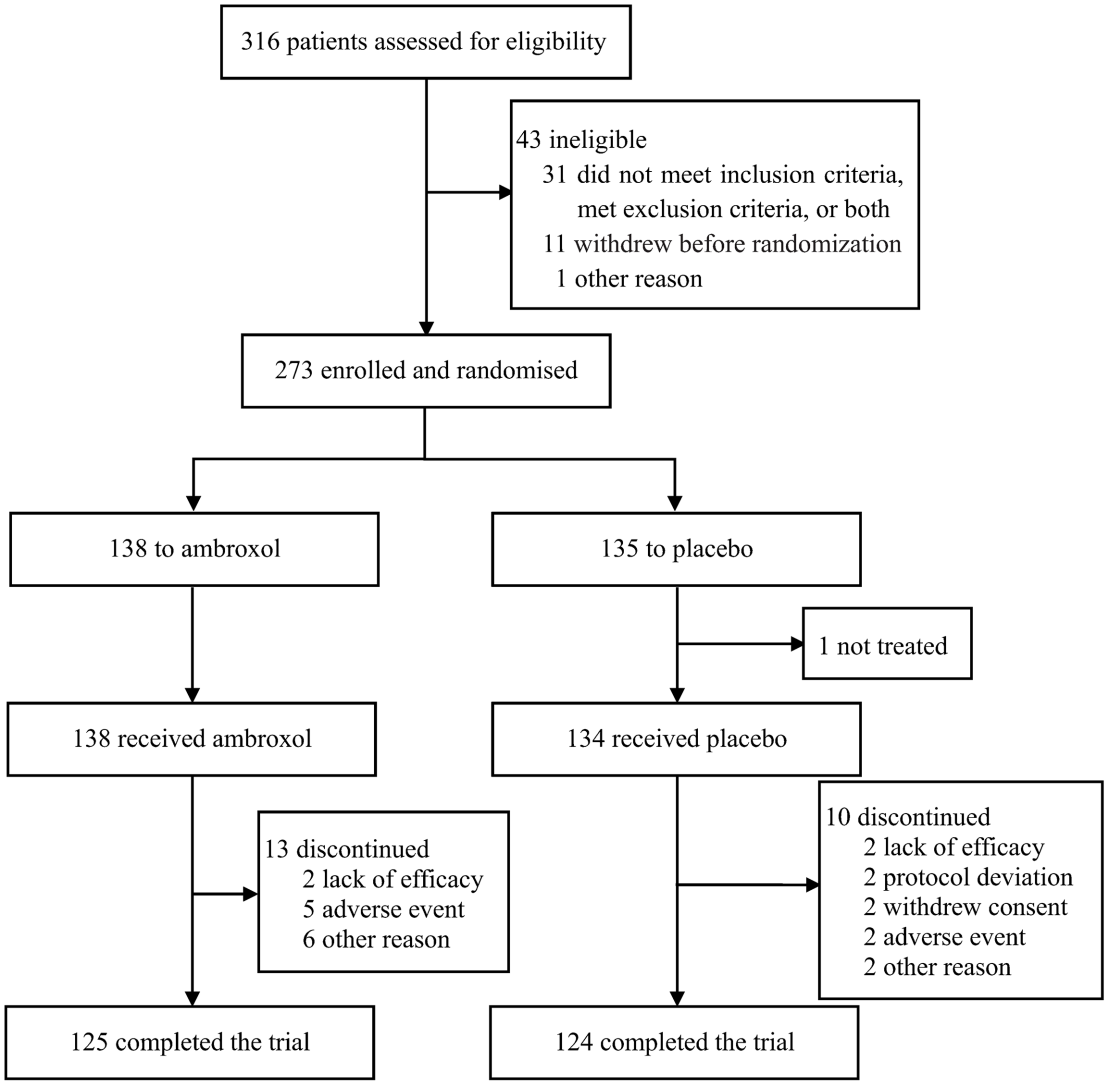
Trial profile.

Between 10 April 2018 and 23 November 2020, 316 patients were recruited and assessed for eligibility, of whom 273 were randomly assigned to ambroxol (*n* = 138) or placebo (*n* = 135). Efficacy data were not available for one patient in the placebo group, thus, 272 patients were included in the intention-to-treat and safety analysis. Of these patients, 125 patients (90.6%) in the ambroxol group and 124 patients (91.9%) in the placebo group completed the assessment (Figure 1).

Baseline demographics and disease characteristics are presented in Table 2, which were well balanced between the two groups. The mean ages were 56.3 and 57.3, and male proportions were 51.5 and 53.7% for the two groups, respectively. The most common reasons for LRTIs were community-acquired pneumonia, acute exacerbation of COPD, and bronchiectasis. Sputum property score, cough intensity, and expectoration difficulty were relatively severer, while expectoration volume in 24 h was relatively smaller in both groups.

**Table 2:** Baseline characteristics.

	Ambroxol (*n* = 138)	Placebo (*n* = 134)
Age, years	56.3 ± 15.5	57.3 ± 15.2
Sex, male	71(51.5)	72(53.7)
Height, cm	164.5 ± 7.7	165.0 ± 7.7
Weight, kg	62.1 ± 12.7	61.3 ± 11.3
Disease		
Pneumonia	55(39.9)	63(47.0)
AECOPD	32(23.2)	27(20.2)
Acute exacerbation of bronchiectasis	15(10.9)	15(11.2)
Other	36(26.1)	29(21.6)
Allergy history	26(18.8)	29(21.6)
Disease or surgical history	106(76.8)	97(72.4)
Sputum property score	2.5 ± 0.5	2.5 ± 0.5
Cough intensity	2.0 ± 0.6	1.9 ± 0.7
Expectoration difficulty	2.4 ± 0.5	2.5 ± 0.5
Expectoration volume in 24 h	0.7 ± 0.6	0.7 ± 0.6
Short acting β-2-agonists in the form of aerosol or dry powder	15(10.9)	16(11.9)

Data are mean ± SD, *n* (%), or median (IQR). There are no significant differences between the two groups at the baseline. AECOPD, acute exacerbation of chronic obstructive pulmonary disease.

### 3.2. Efficacy outcomes

**Figure 2:**
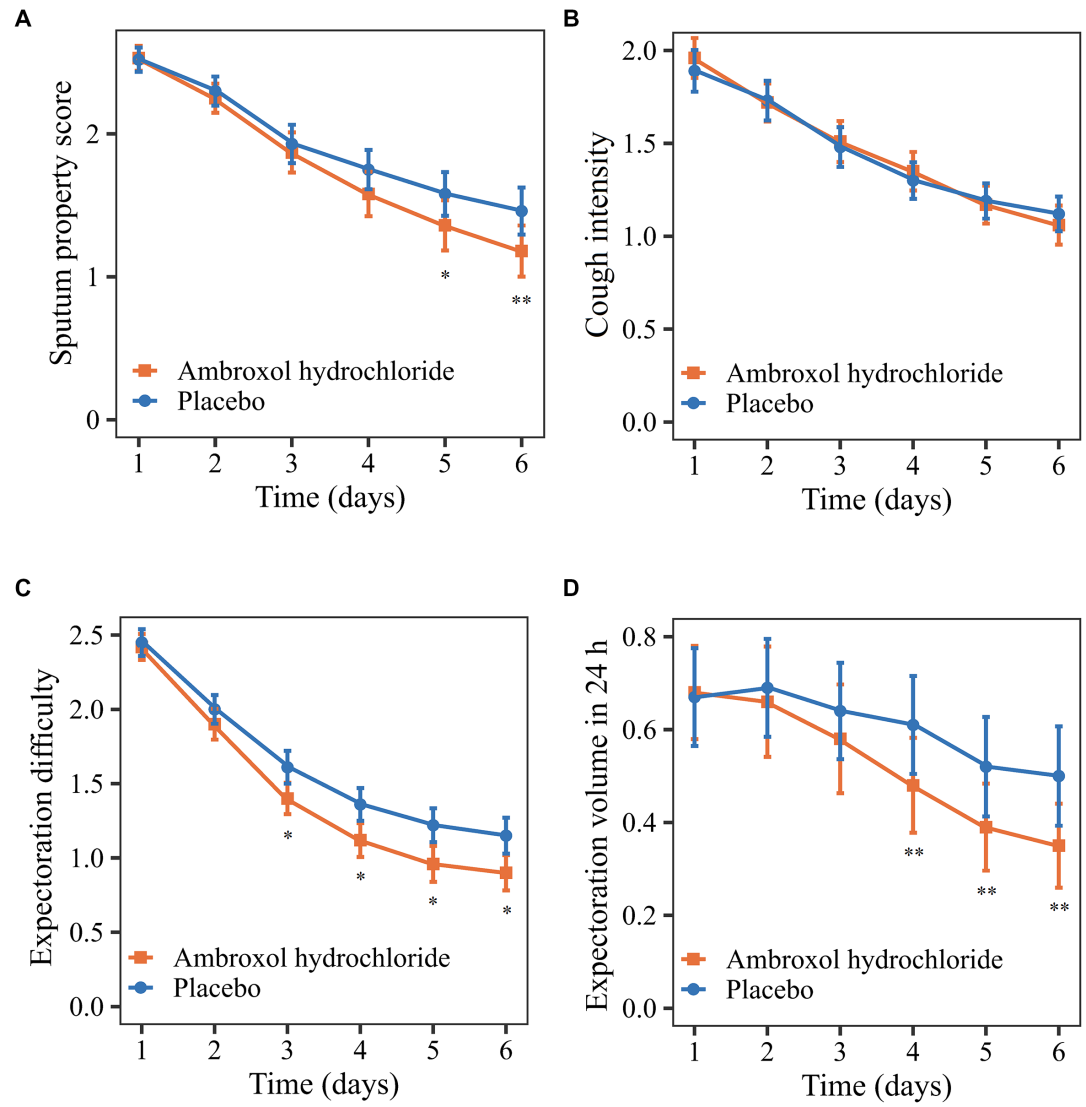
Comparison of primary and secondary outcomes from the baseline to 24 h after treatment. **(A)** Sputum property score. **(B)** Cough intensity. **(C)** Expectoration difficulty. **(D)** Expectoration volume in 24 h. ^*^p < 0.10; ^**^p < 0.05.

The mean change from the baseline to 24 h after treatment in sputum property score was −1.35 (*p* < 0.0001) for the ambroxol group and − 1.06 (*p* < 0.0001) for the placebo group (Table 3). Patients who received inhaled ambroxol had a significantly greater reduction in the sputum property score at 24 h after treatment than did patients who received inhalation of placebo (difference: −0.29; 95% CI: −0.53 to −0.05; *p*: 0.0215; Table 3; Figure 2A). The interaction between the center and the treatment group was not significant (Supplementary Table 2). After adjusting for the baseline sputum property score and the center, the effect of the intervention on the primary efficacy outcome was also statistically significant (Table 4).

**Table 3:** Changes between the baseline and 24 h after treatment in efficacy outcomes.

	Ambroxol (*n* = 138)	Placebo (*n* = 134)	Treatment difference (95% CI)	*p* value
Primary outcome				
Sputum property score	−1.35 ± 1.06	−1.06 ± 0.96	−0.29 (−0.53, −0.05)	0.0215
Secondary outcomes				
Cough intensity	−0.91 ± 0.74	−0.77 ± 0.72	−0.14 (−0.33, 0.04)	0.1231
Expectoration difficulty	−1.50 ± 0.89	−1.31 ± 0.86	−0.20 (−0.42, 0.02)	0.0791
Expectoration volume in 24 h	−0.34 ± 0.60	−0.16 ± 0.64	−0.18 (−0.34, −0.03)	0.0166

**Table 4:** Effect of treatment on the primary efficacy outcome after adjusting for centers and the baseline sputum property score.

Variables	Wald	*p* value
Treatment group	7.97	0.005
Center	44.80	<0.001
Baseline sputum property score	4.45	0.035

Regarding the secondary outcomes, the mean reductions in cough intensity and expectoration difficulty from the baseline to 24 h after treatment in the ambroxol group were greater than that in the placebo group although the differences were not significant (*p* > 0.05; Table 3, Figures 2B,C). Patients in the ambroxol group had a significantly greater reduction in expectoration volume in 24 h compared with placebo (difference: −0.18; 95% CI: −0.34 to −0.03; *p*: 0.0166; Table 3; Figure 2D).

### 3.3. Safety outcomes

The proportions of patients reporting adverse events were 55 (39.9%) of 138 with ambroxol and 50 (37.3%) of 134 with placebo (Table 5). There were four serious adverse events during the study, one (0.7%) reported in the ambroxol group (respiratory failure) and three (2.2%) reported in the placebo group (lung adenocarcinoma, small cell lung cancer, and pulmonary tuberculosis). There were no deaths reported in this study.

**Table 5:** Summary of adverse events in the safety population.

	Ambroxol (*n* = 138)	Placebo (*n* = 134)	*p* value
Any adverse events	55(39.9)	50(37.3)	0.709
Serious adverse events	1(0.7)	3(2.2)	0.365
Adverse events leading to dose modification, measure taken or study discontinuation	31(22.5)	30(22.4)	1.000
Adverse events leading to study discontinuation	6(4.4)	3(2.2)	0.501
Adverse events in at least 1% of participants			
ALT increased	4(2.9)	5(3.7)	0.747
AST increased	2(1.5)	3(2.2)	0.681
WBC decreased	2(1.5)	1(0.8)	1.000
WBC increased	1(0.7)	2(1.5)	0.618
Urine RBC positive	2(1.5)	1(0.8)	1.000
NEU increased	0(0.0)	2(1.5)	0.242
Cough	2(1.5)	4(3.0)	0.442
Hemoptysis	2(1.5)	1(0.8)	1.000
Dry throat	2(1.5)	2(1.5)	1.000
Mycoplasma infection	3(2.2)	3(2.2)	1.000
Upper respiratory tract infection	1(0.7)	2(1.5)	0.618
Rhinitis	0(0.0)	2(1.5)	0.242
Infectious pneumonia	2(1.5)	0(0.0)	0.498
Chlamydia infection	0(0.0)	2(1.5)	0.242
Dry mouth	3(2.2)	3(2.2)	1.000
Nausea	2(1.5)	3(2.2)	0.681
Hypokalemia	1(0.7)	4(3.0)	0.209
Chest discomfort	2(1.5)	2(1.5)	1.000
Dizzy	2(1.5)	3(2.2)	0.681
Hepatic steatosis	3(2.2)	1(0.8)	0.622
Thyroid cyst	0(0.0)	2(1.5)	0.242
Anemia	2(1.5)	3(2.2)	0.681
Pruritus	2(1.5)	0(0.0)	0.498

Table 5 shows 23 kinds of adverse events reported by more than 1% of patients in the ambroxol or placebo groups. The most common adverse events were the upper respiratory tract symptoms and increased aminotransferases. The differences in these adverse events were not statistically significant between the two groups. There were also no clinically meaningful differences in other vital signs, physical examination, laboratory evaluation (routine blood test, routine urine test, hepatic function, and renal function), and electrocardiogram between the two groups (Supplementary Table 3).

## 4. Discussion

The findings of this multicenter, randomized, controlled trial showed that inhaled ambroxol hydrochloride solution twice daily was significantly more effective than a placebo in reducing the sputum property score in patients with mucopurulent sputum due to LRTIs or acute exacerbation of chronic respiratory diseases, reducing 0.29 points more than placebo. Inhaled ambroxol also significantly reduced more expectoration volume in 24 h compared with the placebo. However, significant between-group differences were not observed in cough intensity and expectoration difficulty although the mean reduction in these secondary outcomes tended to be greater in the ambroxol group than in the placebo group. The safety of inhaled ambroxol was comparable to placebo.

Theoretically, ambroxol could have a favorable influence on the sputum clearance of patients with mucopurulent sputum. Previous studies have proved that the main pharmacological effect of ambroxol was stimulation of surfactant production, associated with effective mucokinetic and mucolytic activities (25). According to previous *in vitro* and animal models, ambroxol could increase mucociliary clearance by stimulating mucociliary activity and increasing ciliary beat frequency (26). In addition, antioxidation, anti-inflammatory, and local anesthetic of ambroxol were also reported in some studies (18). Pharmacokinetic studies showed that the tracheal administration of ambroxol could achieve a higher local activity and longer effect compared with intravenous injection (27).

*The post hoc* analysis found that the changes in efficacy outcomes were different during the treatment. The early changes in the sputum property score in the two groups were similar, while the score of the ambroxol group decreased more significantly after a period of treatment (Figure 2A). The early efficacy may be attributed to the dilution of sputum and lubrication of the airway by inhaled solution (28). The difference in the later treatment effect may be caused by the pharmacological effect of ambroxol (18). The expectoration difficulty decreased rapidly in the early phase, while the expectoration volume decreased rapidly in the late phase of treatment (Figures 2C,D). The possible reason was that the accumulated sputum was expectorated due to the reduced expectoration difficulty in the early phase of treatment, resulting in the increase of expectoration volume.

According to a previous review, oral ambroxol was well-tolerated during short-term and long-term treatments and showed no differences in adverse events compared with placebo (29). The adverse events were all mild and self-limiting, mainly including nausea, vomiting, skin rash, insomnia, dry mouth, pyrosis, and chest tightness (22). Unlike previous studies of oral ambroxol, the increase in ALT and AST were the most frequently reported adverse events in this study, which occurred in both the ambroxol group and placebo group. The research team considered that it was difficult to judge the correlation between the increases and ambroxol inhalation based on the current evidence. These increases may be caused by diseases or concomitant medications. For example, two patients used cefotaxime sodium, mezlocillin sodium, sulbactam sodium, or levofloxacin hydrochloride for injection, which may lead to an increase in ALT and AST. Therefore, further post-marketing studies were needed to evaluate the safety of ambroxol inhalation.

This clinical trial had several limitations. This study was conducted in an inpatient population, making the generalizability of the findings to a broad population in the outpatient clinic and community, where there were more confounding factors that are challenging. Due to the limited sample size and short duration, it was difficult to confirm some adverse events and long-term effects, which needed to be evaluated by post-marketing studies.

In conclusion, compared with a placebo, inhaled ambroxol hydrochloride solution twice daily was more effective in sputum clearance in patients with mucopurulent sputum due to LRTIs or acute exacerbation of chronic respiratory diseases. Ambroxol inhalation could reduce sputum viscosity, expectoration difficulty, and volume, without increasing adverse events. Therefore, data from this study supported the view that inhaled ambroxol was safe and effective in adult patients with mucopurulent sputum.

## Data availability statement

The raw data supporting the conclusions of this article will be made available by the authors, without undue reservation.

## Ethics statement

The studies involving human participants were reviewed and approved by ethics committee of First Affiliated Hospital of Guangzhou Medical University. The patients/participants provided their written informed consent to participate in this study.

## Author contributions

ZZ: investigation, conceptualization, methodology, and writing—original draft. KY: formal analysis, investigation, writing—original draft, and visualization. NL, XF, HH, HC, PX, JW, ML, YT, FZ, SX, XY, JHa, BY, BJ, GP, YS, MK, HS, HX, and YP: investigation, data curation, and writing—reviewing and editing. JHe: formal analysis, methodology, and writing—reviewing and editing. RC: investigation, funding acquisition, project administration, and writing—reviewing and editing. All authors contributed to the article and approved the submitted version.

## Funding

This research was supported by Yingu Pharmaceutical Co Ltd.

## Conflict of interest

Author YP was employed by the company Yingu Pharmaceutical Co Ltd. The remaining authors declare that the research was conducted in the absence of any commercial or financial relationships that could be construed as a potential conflict of interest.

The authors declare that this study received funding from Yingu Pharmaceutical Co Ltd. The funder was not involved in the study design, collection, analysis, interpretation of data, the writing of this article or the decision to submit it for publication.

## Publisher’s note

All claims expressed in this article are solely those of the authors and do not necessarily represent those of their affiliated organizations, or those of the publisher, the editors and the reviewers. Any product that may be evaluated in this article, or claim that may be made by its manufacturer, is not guaranteed or endorsed by the publisher.
