# Supplementary material for: Evaluation of safety and efficacy of inhaled ambroxol in hospitalized adult patients with mucopurulent sputum and expectoration difficulty
Source: Front Med (Lausanne). 2023 May 25;10:1182602. doi: 10.3389/fmed.2023.1182602 (PMC10248402; doi:10.3389/fmed.2023.1182602)
Supplement: Supplementary file 1 [file Data_Sheet_1.docx]

Supplementary Material

Evaluation of safety and efficacy of inhaled ambroxol in hospitalized adult patients with mucopurulent sputum and expectoration difficulty

Zeguang Zheng^1, †^, Kai Yang^2, †^, Ni Liu^1^, Xiuhua Fu^3^, Huijie He^4^, Hong Chen^5^, Peijun Xu^6^, Jing Wang^7^, Maofeng Liu^8^, Yuling Tang^9^, Fengzi Zhao^10^, Shufeng Xu^11^, Xiaowei Yu^12^, Jichang Han^13^, Bo Yuan^14^, Bin Jia^15^, Guifen Pang^16^, Yantong Shi^17^, Min Kuang^18^, Haiyan Shao^19^, Hao Xiong^20^, Jia He^21^, Yuanyuan Pan^22^, Rongchang Chen^2, *^

*** Correspondence:** Rongchang Chen: [chenrc@vip.163.com](mailto:chenrc@vip.163.com)

# Supplementary Data

**1.1 Inclusion criteria**

Subjects must fulfil all of the following criteria before they can be enrolled:

- men and women aged 18-80 years old.
- Patients with lower respiratory tract infection (e.g. community-acquired pneumonia, acute exacerbation of chronic obstructive pulmonary disease, acute exacerbation of chronic bronchitis, bronchiectasis without hemoptysis) with purulent sputum (sputum property score ≥ 2 points) and expectoration difficulty (expectoration difficulty ≥ 2 points).
- Patients requiring hospitalization during medication.
- Patients who were confirmed to meet the target population through clinical symptoms, signs and laboratory tests.
- The informed consent was signed voluntarily, and the subjects of childbearing age volunteered to take effective contraceptive measures during the trial.

**1.2 Exclusion criteria**

If the subjects met any of the following criteria, they cannot be enrolled in the trial:

- Those who were known to be allergic to the ingredients contained in the study drug.
- Patients with pulmonary tuberculosis, pneumoconiosis, lung cancer, pulmonary interstitial diseases, bronchiectasis with hemoptysis, pulmonary interstitial fibrosis and other severe respiratory diseases.
- Patients with severe primary diseases of cardiovascular, cerebrovascular, liver, kidney and hematopoietic system; Alanine aminotransferase (ALT) or aspartate aminotransferase (AST) were greater than 1.5 times the upper limit of the normal value, or serum creatinine (CR) was greater than the upper limit of the normal value (refer to the normal value range of the laboratory examination of the center); Fever accompanied by clear bacterial infection symptoms, and the white blood cell count was greater than 1.5 times the upper limit of the normal value.
- Patients with alcohol or drug dependence.
- Patients with a history of epilepsy or mental disorders.
- Pregnant or lactating women, or those planning pregnancy (including men and women).
- Those who have participated in or are participating in other clinical trials within 3 months.
- Patients with difficulty in coughing up sputum due to tracheal stenosis (e.g. the history of respiratory tract tumor, foreign body airway obstruction).
- Patients complicated with cerebrovascular accident, cachexia, severe consumptive disease, myasthenia, long-term bed rest, etc., resulting in weak or unable sputum expectoration. Patients who cannot expectorate and needed atomization inhalation or tracheotomy for sputum expectoration.
- Patients who cannot cooperate with inhalation treatment or needed sputum clearance by physiotherapies.
- Other patients considered unsuitable by the investigator.

# Supplementary Tables

Supplementary Table 1. Study procedures.

| **Stage** | **Screening** | **Hospitalization** | | | | | **End of study** |
| --- | --- | --- | --- | --- | --- | --- | --- |
| Visit | Visit 1 | Visit 2  Baseline | Visit 3 | Visit 4 | Visit 5 | Visit 6 | Visit 7 |
| Visit window | Day -1 ~ 0 | Day 1 | Day 2 | Day 3 | Day 4 | Day5 | Day 6 |
| **Baseline characteristics** |  |  |  |  |  |  |  |
| Informed consent | √ |  |  |  |  |  |  |
| Inclusion and exclusion criteria | √ |  |  |  |  |  |  |
| Basic information | √ |  |  |  |  |  |  |
| Disease and treatment history | √ |  |  |  |  |  |  |
| Random allocation |  | √ |  |  |  |  |  |
| Efficacy outcomes |  |  |  |  |  |  |  |
| Sputum property score | √ | √ | √ | √ | √ | √ | √ |
| Cough intensity |  | √ | √ | √ | √ | √ | √ |
| Expectoration difficulty | √ | √ | √ | √ | √ | √ | √ |
| Expectoration volume in 24 h |  | √ | √ | √ | √ | √ | √ |
| **Safety outcomes** |  |  |  |  |  |  |  |
| Physical examination | √ | √ | √ | √ | √ | √ | √ |
| Vital signs | √ | √ | √ | √ | √ | √ | √ |
| Routine blood test | √ |  |  |  |  |  | √ |
| Routine urine test | √ |  |  |  |  |  | √ |
| Hepatic function | √ |  |  |  |  |  | √ |
| Renal function | √ |  |  |  |  |  | √ |
| Electrocardiogram | √ |  |  |  |  |  | √ |
| **Examination for screening** |  |  |  |  |  |  |  |
| Chest x-ray | √ |  |  |  |  |  |  |
| Urine pregnancy test ^*^ | √ |  |  |  |  |  | √ |
| **Other** |  |  |  |  |  |  |  |
| Record adverse events |  | √ | √ | √ | √ | √ | √ |
| Record concomitant medication | √ | √ | √ | √ | √ | √ | √ |
| Record atomization record |  | √ | √ | √ | √ | √ |  |

Note: ^*^ It was only for premenopausal women and should be tested within 24 hours before the first medication. Urine pregnancy test should be performed when secondary amenorrhea occurred for more than 1 week.

Supplementary Table 2. The logistic model included the interaction between center and treatment group.

| Variables | Wald | P value |
| --- | --- | --- |
| Treatment group | 0.00 | 0.993 |
| Center | 45.87 | <0.001 |
| Baseline sputum property score | 2.23 | 0.136 |
| Center × Treatment group | 22.94 | 0.193 |

Supplementary Table 3. The detailed list of adverse events in safety population.

|  | Ambroxol (n = 138) | Placebo (n = 134) | P value |
| --- | --- | --- | --- |
| **Laboratory evaluation** | 14(10.1) | 12(9.0) | 0.838 |
| Alanine aminotransferase increased | 4(2.9) | 5(3.7) | 0.747 |
| Aspartate aminotransferase increased | 2(1.5) | 3(2.2) | 0.681 |
| White blood cell count decreased | 2(1.5) | 1(0.8) | 1.000 |
| White blood cell count increased | 1(0.7) | 2(1.5) | 0.618 |
| Urine red blood cell positive | 2(1.5) | 1(0.8) | 1.000 |
| Abnormal carbon dioxide binding | 1(0.7) | 1(0.8) | 1.000 |
| Neutrophil count decreased | 1(0.7) | 1(0.8) | 1.000 |
| Neutrophil count increased | 0(0.0) | 2(1.5) | 0.242 |
| C-reactive protein increased | 1(0.7) | 0(0.0) | 1.000 |
| γ-glutamyltransferase increased | 1(0.7) | 0(0.0) | 1.000 |
| Hematocrit increased | 0(0.0) | 1(0.8) | 0.493 |
| Lymphocyte count increased | 1(0.7) | 0(0.0) | 1.000 |
| Urine protein positive | 1(0.7) | 0(0.0) | 1.000 |
| Urine crystallization | 1(0.7) | 0(0.0) | 1.000 |
| Blood in urine | 1(0.7) | 0(0.0) | 1.000 |
| Abnormal electrocardiogram | 0(0.0) | 1(0.8) | 0.493 |
| Blood albumin decreased | 0(0.0) | 1(0.8) | 0.493 |
| Blood creatinine increased | 1(0.7) | 0(0.0) | 1.000 |
| Total protein decreased | 0(0.0) | 1(0.8) | 0.493 |
| **Respiratory system, chest and mediastinal diseases** | 14(10.1) | 8(6) | 0.267 |
| Cough | 2(1.5) | 4(3) | 0.442 |
| Hemoptysis | 2(1.5) | 1(0.8) | 1.000 |
| Dry throat | 2(1.5) | 2(1.5) | 1.000 |
| Infectious pneumonia | 2(1.5) | 0(0.0) | 0.498 |
| Bronchial hemorrhage | 1(0.7) | 1(0.8) | 1.000 |
| Paranasal cyst | 1(0.7) | 0(0.0) | 1.000 |
| Nasal congestion | 1(0.7) | 0(0.0) | 1.000 |
| Cyanosis | 1(0.7) | 0(0.0) | 1.000 |
| Lung mass | 0(0.0) | 1(0.8) | 0.493 |
| Shortness of breath | 1(0.7) | 0(0.0) | 1.000 |
| Dyspnea | 1(0.7) | 0(0.0) | 1.000 |
| Respiratory failure | 1(0.7) | 0(0.0) | 1.000 |
| Cystic lung disease | 1(0.7) | 0(0.0) | 1.000 |
| Hypoxia | 1(0.7) | 0(0.0) | 1.000 |
| Sleep apnea syndrome | 1(0.7) | 0(0.0) | 1.000 |
| Wheezing | 0(0.0) | 1(0.8) | 0.493 |
| **Infectious diseases** | 8(5.8) | 11(8.2) | 0.483 |
| Mycoplasma infection | 3(2.2) | 3(2.2) | 1.000 |
| Upper respiratory tract infection | 1(0.7) | 2(1.5) | 0.618 |
| Rhinitis | 0(0.0) | 2(1.5) | 0.242 |
| Infectious pneumonia | 2(1.5) | 0(0.0) | 0.498 |
| Urinary tract infection | 1(0.7) | 1(0.8) | 1.000 |
| Chlamydia infection | 0(0.0) | 2(1.5) | 0.242 |
| Nasosinusitis | 0(0.0) | 1(0.8) | 0.493 |
| Pulmonary tuberculosis | 0(0.0) | 1(0.8) | 0.493 |
| Conjunctivitis | 1(0.7) | 0(0.0) | 1.000 |
| Helicobacter infection | 1(0.7) | 0(0.0) | 1.000 |
| Syphilis | 1(0.7) | 0(0.0) | 1.000 |
| Pharyngitis | 1(0.7) | 0(0.0) | 1.000 |
| **Gastrointestinal diseases** | 7(5.1) | 8(6.0) | 0.796 |
| Dry mouth | 3(2.2) | 3(2.2) | 1.000 |
| Nausea | 2(1.5) | 3(2.2) | 0.681 |
| Abdominal discomfort | 0(0.0) | 1(0.8) | 0.493 |
| Abdominal pain | 1(0.7) | 0(0.0) | 1.000 |
| Diarrhea | 1(0.7) | 0(0.0) | 1.000 |
| Colitis | 0(0.0) | 1(0.8) | 0.493 |
| Vomit | 1(0.7) | 0(0.0) | 1.000 |
| Prolapse of stomach | 0(0.0) | 1(0.8) | 0.493 |
| Gastric polyp | 1(0.7) | 0(0.0) | 1.000 |
| Gastritis | 0(0.0) | 1(0.8) | 0.493 |
| **Metabolic and nutritional diseases** | 3(2.2) | 5(3.7) | 0.496 |
| Hypokalemia | 1(0.7) | 4(3.0) | 0.209 |
| Hypoproteinemia | 1(0.7) | 1(0.8) | 1.000 |
| Hyperlipidemia | 1(0.7) | 1(0.8) | 1.000 |
| Hypocalcemia | 0(0.0) | 1(0.8) | 0.493 |
| Hypochloremia | 0(0.0) | 1(0.8) | 0.493 |
| **Systemic diseases and reactions at the administration site** | 4(2.9) | 6(4.5) | 0.536 |
| Chest discomfort | 2(1.5) | 2(1.5) | 1.000 |
| Fever | 1(0.7) | 1(0.8) | 1.000 |
| Edema at intubation site | 0(0.0) | 1(0.8) | 0.493 |
| Chest pain | 0(0.0) | 1(0.8) | 0.493 |
| **Various neurological diseases** | 4(2.9) | 4(3.0) | 1.000 |
| Dizzy | 2(1.5) | 3(2.2) | 0.681 |
| Headache | 1(0.7) | 1(0.8) | 1.000 |
| Dysgeusia | 1(0.7) | 0(0.0) | 1.000 |
| **Hepatobiliary diseases** | 3(2.2) | 2(1.5) | 1.000 |
| Hepatic steatosis | 3(2.2) | 1(0.8) | 0.622 |
| Hepatic sarcoidosis | 0(0.0) | 1(0.8) | 0.493 |
| **Musculoskeletal and connective tissue diseases** | 2(1.5) | 3(2.2) | 0.681 |
| Arthralgia | 0(0.0) | 1(0.8) | 0.493 |
| Periarthritis | 0(0.0) | 1(0.8) | 0.493 |
| Musculoskeletal discomfort | 1(0.7) | 0(0.0) | 1.000 |
| Connective tissue disease | 1(0.7) | 0(0.0) | 1.000 |
| Protrusion of intervertebral disc | 0(0.0) | 1(0.8) | 0.493 |
| **Benign, malignant and unknown tumors (including cystic and polypoid)** | 2(1.5) | 3(2.2) | 0.681 |
| Colonic adenoma | 0(0.0) | 1(0.8) | 0.493 |
| Lung adenocarcinoma | 0(0.0) | 1(0.8) | 0.493 |
| Small cell carcinoma | 0(0.0) | 1(0.8) | 0.493 |
| Lipoma | 1(0.7) | 0(0.0) | 1.000 |
| Uterine leiomyoma | 1(0.7) | 0(0.0) | 1.000 |
| **Endocrine system diseases** | 1(0.7) | 3(2.2) | 0.365 |
| Thyroid cyst | 0(0.0) | 2(1.5) | 0.242 |
| Thyroid nodule | 1(0.7) | 1(0.8) | 1.000 |
| Thyroncus | 0(0.0) | 1(0.8) | 0.493 |
| **Diseases of blood and lymphatic system** | 2(1.5) | 3(2.2) | 0.681 |
| Anemia | 2(1.5) | 3(2.2) | 0.681 |
| **Heart disease** | 2(1.5) | 2(1.5) | 1.000 |
| Tricuspid valve disease | 0(0.0) | 1(0.8) | 0.493 |
| Ventricular extrasystole | 1(0.7) | 0(0.0) | 1.000 |
| Ventricular arrhythmia | 0(0.0) | 1(0.8) | 0.493 |
| First degree atrioventricular block | 1(0.7) | 0(0.0) | 1.000 |
| **Psychosis** | 2(1.5) | 1(0.8) | 1.000 |
| Anxious | 1(0.7) | 0(0.0) | 1.000 |
| Insomnia | 1(0.7) | 0(0.0) | 1.000 |
| Sleep disorder | 0(0.0) | 1(0.8) | 0.493 |
| **Diseases of kidney and urinary system** | 1(0.7) | 2(1.5) | 0.618 |
| Renal cyst | 1(0.7) | 1(0.8) | 1.000 |
| Frequency of urinatior | 0(0.0) | 1(0.8) | 0.493 |
| **Reproductive system and breast diseases** | 1(0.7) | 2(1.5) | 0.618 |
| Benign prostatic hyperplasia | 1(0.7) | 1(0.8) | 1.000 |
| Hyperplasia of mammary glands | 0(0.0) | 1(0.8) | 0.493 |
| **Injuries, poisoning and surgical complications** | 0(0.0) | 1(0.8) | 0.493 |
| Inflammation of gastric relict | 0(0.0) | 1(0.8) | 0.493 |
| Incision site inflammation | 0(0.0) | 1(0.8) | 0.493 |
| **Congenital and hereditary diseases** | 2(1.5) | 0(0.0) | 0.498 |
| Accessory spleen | 1(0.7) | 0(0.0) | 1.000 |
| Congenital heart disease | 1(0.7) | 0(0.0) | 1.000 |
| **Immune system diseases** | 1(0.7) | 1(0.8) | 1.000 |
| Hypersensitivity | 0(0.0) | 1(0.8) | 0.493 |
| Autoimmune diseases | 1(0.7) | 0(0.0) | 1.000 |
| **Skin and subcutaneous diseases** | 2(1.5) | 0(0.0) | 0.498 |
| Pruritus | 2(1.5) | 0(0.0) | 0.498 |
| **Vascular and lymphatic diseases** | 2(1.5) | 0(0.0) | 0.498 |
| Hypotension | 1(0.7) | 0(0.0) | 1.000 |
| Vasculitis | 1(0.7) | 0(0.0) | 1.000 |
| **Surgical and medical operations** | 1(0.7) | 0(0.0) | 1.000 |
| Diabetes treatment | 1(0.7) | 0(0.0) | 1.000 |
| **Eye diseases** | 1(0.7) | 0(0.0) | 1.000 |
| Eyelid edema | 1(0.7) | 0(0.0) | 1.000 |
